# Editorial: Beyond therapeutic innovation in genitourinary cancers: the unmet need for biologically driven patient selection

**DOI:** 10.3389/fimmu.2026.1859297

**Published:** 2026-05-06

**Authors:** Brigida Anna Maiorano, Linda Cerbone, Carlo Messina, Pasquale Rescigno, Giandomenico Roviello

**Affiliations:** 1 Department of Medical Oncology, Comprehensive Cancer Center, Istituto di Ricovero e Cura a Carattere Scientifico (IRCCS) San Raffaele, Milan, Italy; 2 Oncologia Medica 1, Istituto di Ricovero e Cura a Carattere Scientifico (IRCCS) Regina Elena National Cancer Institute, Rome, Italy; 3 Oncology Unit, Azienda Ospedaliera di Rilievo Nazionale e di Alta Specializzazione (A.R.N.A.S.) Civico, Palermo, Italy; 4 Translational and Clinical Research Institute, The Newcastle upon Tyne Hospitals National Health Service (NHS) Foundation Trust, Newcastle Upon Tyne, United Kingdom; 5 Department of Health Sciences, Section of Clinical Pharmacology and Oncology, University of Florence, Florence, Italy

**Keywords:** biomarkers, genitourinary cancers, immunotherapy, metabolic reprogramming, precision oncology, tumor microenvironment

Over the past decade, the therapeutic landscape of genitourinary malignancies has undergone a profound transformation. The introduction of immune checkpoint inhibitors (ICIs), alongside antibody–drug conjugates, tyrosine kinase inhibitors, and other targeted therapies, has significantly expanded treatment options across multiple disease settings. However, these advances have not translated into uniform clinical benefit. While substantial improvements have been observed in renal cell carcinoma (RCC) and urothelial carcinoma (UC), other tumors, such as prostate and testicular cancers, continue to exhibit limited sensitivity to immunotherapeutic strategies.

This evolving scenario has generated a central paradox in modern genitourinary oncology: therapeutic innovation is rapidly accelerating, yet our ability to accurately identify patients who will benefit from these treatments remains limited. The Research Topic “Immunotherapy and novel agents in genito-urinary tumors” was conceived within this context, aiming to explore not only novel therapeutic strategies but also the biological determinants of response and resistance, with a particular emphasis on biomarker development and translational insights.

A prominent theme emerging from this Research Topic is the expanding - but still insufficiently refined - role of biomarkers in guiding clinical decision-making. Several studies highlight the prognostic and predictive value of both tissue-based and circulating biomarkers in UC, including immunohistochemical panels, inflammation-related indices, and cytokine-based signatures, all of which demonstrate consistent associations with survival outcomes and response to ICIs (Ren et al., Sharif et al., Szeles et al., Tang et al., Maiorano et al.). Similarly, systematic evaluations of circulating inflammatory markers reinforce the interplay between systemic inflammation and antitumor immunity, underscoring their potential clinical utility while also revealing their current limitations in terms of standardization and reproducibility (Ren et al., Szeles et al.). Collectively, these findings emphasize that, despite their accessibility and clinical appeal, currently available biomarkers remain heterogeneous and insufficient to fully capture the complexity of treatment response.

Beyond conventional biomarkers, this Research Topic underscores the critical role of tumor biology and microenvironmental complexity in shaping therapeutic outcomes. Multi-omics, single-cell, and spatial analyses reveal a highly heterogeneous tumor ecosystem, in which immune infiltration, angiogenesis, and stromal components interact in a dynamic and context-dependent manner (Wang et al., Cao et al., Song et al.). For instance, angiogenesis-related pathways and genes such as Nidogen-2 (NID2) appear to influence immune infiltration and immunotherapy responsiveness, while single-cell transcriptomic analyses in renal cell carcinoma uncover diverse functional states of mast cells within the tumor microenvironment (Cao et al., Song et al.). At the same time, mechanistic studies in prostate cancer provide insights into tumor-intrinsic signaling pathways, such as the Ca²^+^/Calcium-Calmodulin-Dependent Protein Kinase Kinase 2 (CAMKK2)/AMP-Activated Protein Kinase (AMPK)/Androgen Receptor (AR) axis, that may contribute to disease progression and therapeutic resistance, particularly in advanced settings (Jia et al.).

Importantly, several contributions extend this framework by integrating metabolic and epigenetic layers of regulation. Metabolic reprogramming emerges as a key determinant of tumor progression and therapeutic sensitivity, as illustrated by studies on fatty acid metabolism and lactylation-related gene signatures, which identify Peroxiredoxin-1 (PRDX1) and 7-Dehydrocholesterol Reductase (DHCR7) as central regulators of tumor aggressiveness, chemoresistance, and immunotherapy efficacy (Wang et al., Zhao et al.). These findings suggest that metabolic pathways are not merely passive hallmarks of cancer, but active drivers of immune modulation and treatment response, linking tumor-intrinsic biology with the surrounding microenvironment. In parallel, regulatory molecules such as microRNAs further highlight the complexity of post-transcriptional control mechanisms in urological malignancies, reinforcing their potential as both biomarkers and therapeutic targets (Li et al.).

While the majority of contributions focus on bladder cancer - reflecting the rapid evolution of therapeutic strategies in this disease - the Research Topic also captures the broader heterogeneity of genitourinary malignancies. Case reports and exploratory studies illustrate the potential of combination strategies and precision oncology approaches, including the use of ICIs with antiangiogenic agents, chemotherapy combinations, and targeted therapies in rare molecular contexts such as Anaplastic Lymphoma Kinase (ALK)-rearranged tumors (Wang et al., Ning et al., Ren et al.). In addition, emerging evidence in less common malignancies, such as penile and testicular tumors, highlights both the opportunities and the persistent unmet clinical needs in these settings (Chen et al.,Benjamin and Hsu).

Taken together, the studies included in this Research Topic illustrate a field in transition. The paradigm is shifting from a treatment-centered approach toward a more integrated, biology-driven model, in which therapeutic decisions are increasingly informed by molecular, immune, and metabolic features. However, this transition remains incomplete. Current biomarkers are often static and fragmented, and they fail to fully reflect the dynamic nature of tumor evolution and treatment response.

Future progress in genitourinary oncology will therefore depend on the development of integrative and dynamic predictive models that combine multi-omics data, liquid biopsy approaches, and advanced computational tools. Rather than relying on single biomarkers, a multidimensional characterization of tumors and their microenvironment will be required to guide treatment selection, optimize sequencing strategies, and ultimately improve patient outcomes.

In this context, the integration of metabolic, immune, and molecular layers represents a promising direction for the field. The challenge ahead is not only to develop new therapies, but to understand, with increasing precision, which patients are most likely to benefit from them. Collectively, the contributions included in this Research Topic provide important insights into this evolving landscape and highlight the path toward a more personalized and biologically informed approach to genitourinary cancer treatment.

